# Mesenchymal stem cells stabilize the blood–brain barrier through regulation of astrocytes

**DOI:** 10.1186/s13287-015-0180-4

**Published:** 2015-09-29

**Authors:** Hyun Jung Park, Jin Young Shin, Ha Na Kim, Se Hee Oh, Sook K. Song, Phil Hyu Lee

**Affiliations:** Department of Neurology and Brain Research Institute, Yonsei University College of Medicine, 250 Seongsanno, Seodaemun-gu, Seoul 120-752 South Korea; Severance Biomedical Science Institute, Yonsei University College of Medicine, 250 Seongsanno, Seodaemun-gu, Seoul 120-752 South Korea; Department of Neurology, Jeju University College of Medicine, 102 Jejudaehak-ro, Jeju, 690-756 South Korea

## Abstract

**Introduction:**

The blood–brain barrier (BBB) protects the brain against potentially neurotoxic molecules in the circulation, and loss of its integrity may contribute to disease progression in neurodegenerative conditions. Recently, the active role of reactive astrocytes in BBB disruption has become evident in the inflamed brain. In the present study, we investigated whether mesenchymal stem cell (MSC) treatment might modulate reactive astrocytes and thus stabilize BBB integrity through vascular endothelial growth factor A (VEGF-A) signaling in inflammatory conditions.

**Methods:**

For the inflamed brain, we injected LPS using a stereotaxic apparatus and MSCs were injected into the tail vein. At 6 hours and 7 days after LPS injection, we analyzed modulatory effects of MSCs on the change of BBB permeability through VEGF-A signaling using immunochemistry and western blot. To determine the effects of MSCs on VEGF-A-related signaling in cellular system, we had used endothelial cells treated with VEGF-A and co-cultured astrocyte and BV 2 cells treated with lipopolysaccharide (LPS) and then these cells were co-cultured with MSCs.

**Results:**

In LPS-treated rats, MSCs restored Evans blue infiltration and the number of endothelial-barrier antigen (EBA) and P-glycoprotein (p-gp)-expressing cells, which were significantly altered in LPS-treated animals. Additionally, MSC administration following LPS treatment markedly increased the density of astrocytic filaments around vessels and reversed LPS-induced elevations in VEGF-A levels as well as endothelial nitric oxide synthase (eNOS)-dependent downregulation of tight junction proteins in the endothelium. Consequently, MSC treatment reduced neutrophil infiltration and enhanced survival of midbrain dopaminergic neurons in LPS-treated animals. In cellular system, MSC treatment led to a significant reversion of VEGF-A-induced eNOS and tight junction protein expression in endothelial cells, which led to increased EBA expressing cells. Additionally, MSC treatment significantly attenuated LPS-induced increased expressions of IL-1β in microglia and VEGF-A in astrocytes with an increase in IL-10 levels.

**Conclusion:**

The present study indicated that MSCs may stabilize BBB permeability by modulating astrocytic endfeet and VEGF-A signaling, which may be relevant to the treatment of Parkinsonian diseases as a candidate for disease modifying therapeutics.

**Electronic supplementary material:**

The online version of this article (doi:10.1186/s13287-015-0180-4) contains supplementary material, which is available to authorized users.

## Introduction

The blood–brain barrier (BBB) tightly regulates ion balance and nutrient transport, and acts as a protective barrier to shield the central nervous system from potentially neurotoxic molecules in the circulation. The integrity of the BBB is altered in neurodegenerative diseases such as Alzheimer’s disease and Parkinson’s disease (PD), and seems to be influenced by disease severity and duration [1, 2]. BBB dysfunction may therefore contribute to disease progression in neurodegenerative conditions, although the underlying mechanism has not been elucidated fully.

Astrocytes, the most abundant cells in the brain, contact the brain vasculature via their endfeet processes. These contacts consist of endothelial tight junctions, which probably prevent diffusion of toxic materials across the BBB [3]. However, reactive astrocytes in the inflamed brain retract their endfeet from vessels, increasing BBB permeability, and proliferate, giving rise to glial scars [4–6]. Furthermore, reactive astrocytes secrete increased levels of the proangiogenic vascular endothelial growth factor A (VEGF-A) more than basal levels in normal physiological conditions. Many cellular and in vivo studies have demonstrated that inflammation-induced expression of VEGF-A induces BBB breakdown and immune cell infiltration through disrupted tight junctions, accompanied by altered expression of the tight junction proteins [7–9]. The downstream cascade appears to be mediated by endothelial nitric oxide synthase (eNOS); systemic administration of a selective eNOS inhibitor abrogates VEGF-A-induced BBB disruption and protects against neurologic deficits in models of inflammatory disease [9]. Blockade of VEGF-A signaling may therefore be a viable strategy to preserve BBB integrity in neurodegenerative diseases.

Previously, we demonstrated that mesenchymal stem cells (MSCs) protect dopaminergic neurons through anti-inflammatory properties mediated by modulation of microglial activation in animal models of PD [10, 11]. Additionally, in 1-methyl-4-phenyl-1,2,3,6-tetrahydropyridine (MPTP)-induced PD, Chao et al. [12] reported recently that this effect promoted recovery of BBB integrity. In the present study, we investigated whether MSC treatment might modulate reactive astrocytes and thus stabilize BBB integrity through VEGF-A signaling pathways in both cellular and animal models of inflammatory conditions.

## Materials and methods

### Antibodies

Antibodies and staining reagents included mouse anti-tyrosine hydroxylase (TH; 1:2000 dilution for brain tissue, dopaminergic neuron marker, Pel-Freez, Rogers, AR, USA) and rabbit anti-ionized calcium-binding adapter molecule 1 (Iba-1; 1:1000 dilution, activated microglia marker, Wako, Osaka, Japan) anti-GFAP (1:1000 dilution, astrocyte marker, Chemicon, Darmstadt, Germany), anti-myeloperoxidase (MPO; 1:1000 dilution, neutrophil marker, Dako, Glostrup, Denmark), anti-endothelial barrier antigen (EBA; 1:1000 dilution, endothelial cell marker, Sternberger Monoclonals, Lutherville, MD, USA), anti-P-glycoprotein (P-gp; 1:5000 dilution, membranetransporter marker, Becton Dickinson, USA), anti-claudin-5 (CLN-5; 1:500 dilution, tight junction protein marker, Invitrogen, Waltham, MA, USA), anti-endothelial nitric oxide synthase (eNOS, 1:2500 dilution, BD Bioscience, Mississauga, ON, Canada), anti-IL-10 (1:200 dilution, anti-inflammatory marker, Santa Cruz, Santa Cruz, CA, USA), anti-IL-1b (1:200 dilution, inflammatory marker, Santa Cruz), anti-CD 31(1:200 dilution, endothelial cell marker, Nouves), anti- aquaporin 4 (AQP-4; 1:50 dilution, astrocyte endfeet marker, Santa Cruz, Santa Cruz, CA, USA) and mouse anti-nuclear mitotic apparatus protein (NuMA, 1:100 dilution; Calbiochem, San Diego, CA, USA), as well as FITC-labeled tomato lectin (1:1000; Vector Laboratories, Burlingame, CA, USA).

### Animal studies

Male Sprague–Dawley rats (250–270 g) were anesthetized with 10 % chloral hydrate prior to lipopolysaccharide (LPS) injection. LPS (5 μg dissolved in 3 μl) was delivered unilaterally into the left substantia nigra (SN; 5.3 mm posterior, 2.3 mm lateral, 7.7 mm ventral from the bregma) and injected at a rate of 0.2 μl/minute using a 26-gauge Hamilton syringe attached to an automated microinjector using a stereotaxic apparatus [13]. The needle was then left in place for an additional 10 minutes before slow retraction. The control group was injected with phosphate-buffered saline (PBS; 3 μl) at the same position and using the same method. At 4 hours after LPS injection, MSCs (1 × 10^6^ cells in 200 μl) were injected into the tail vein [11]. For tissue preparation, rats were sacrificed at 6 hours, 12 hours, 1 day, 2 days, and 7 days after the LPS injection [14]. Histopathological samples from three groups of rats (control, LPS only, MSC treatment following LPS) were compared. The animal work was approved by the Institutional Animal Care and Use Committee of Yonsei University (approval number: 2013–0379).

### Isolation of MSCs

Bone marrow aspirates (10 ml) were obtained from the iliac crests of human donors. The mononuclear cell layer was isolated by Ficoll–Hypaque, washed in PBS, plated in polystyrene plastic 100 mm culture dishes, and cultivated in low-glucose Dulbecco’s modified Eagle’s medium (DMEM; Gibco-BRL, Grand Island, NY, USA) containing 10 % fetal bovine serum (FBS; Hyclone, Irvine, CA, USA) and 1 % penicillin/streptomycin (Sigma, St. Louis, MO, USA) in a humidified incubator at 37 °C under 5 % CO_2_. Nonadherent cells were removed after 24 hours. When these primary cultures reached 80 % confluence, the cells were harvested using 0.25 % trypsin and subcultured. At passage 6, MSCs were injected into the tail vein or cultured in individual wells of 24-transwell plates (3 × 10^3^ cells/transwell) or six-transwell plates (3 × 10^4^ cells/transwell) 1 day before the experiment. The study protocol and consent form were approved by the Institutional Review Board for Human Investigation of Yonsei University Severance Hospital.

### Extravasation of Evans blue dye

To measure the vascular permeability in the brain, Evans blue dye (EB, 4 % in saline, 3 ml/kg) was intravenously injected. Fifteen minutes later, the rats were anesthetized and perfused. The frozen sections were prepared, and the infiltrated EB was analyzed using a confocal microscope (Carl Zeiss, Oberkochen, Germany).

### Tissue preparation

For immunohistochemistry, rats were perfused with saline solution containing 0.5 % sodium nitrate and heparin (10 U/ml) and fixed with 4 % paraformaldehyde dissolved in 0.1 M PBS (~150 ml/rat) at 12 hours and 7 days after the first drug injection. Brains were removed and post-fixed overnight in buffered 4 % paraformaldehyde at 4 °C and stored in a 30 % sucrose solution for 1–2 days at 4 °C until they sank. The brains were then sectioned into 30 μm coronal slices, which were stored in tissue stock solution (30 % glycerol, 30 % ethylene glycol, 30 % three-times distilled water, 10 % 0.2 M phosphate buffer (PB)) at 4 °C until required. For western blotting, rats were euthanized at 12 hours after the first injection, and the SN were rapidly dissected and frozen at −70 °C. For enzyme-linked immunosorbent assay (ELISA), rats were euthanized at 6 hours, 12 hours, 1 day, 2 days, and 7 days after first injection, and the SN were rapidly dissected and frozen at −70 °C.

### Immunohistochemistry and immunocytochemistry

Coronal brain sections and cocultured bEnd.3 cells were rinsed twice in PBS, incubated in 0.2 % Triton X-100 for 30 minutes at room temperature, and rinsed three times with 0.5 % bovine serum albumin (BSA) in 1× PBS for blocking. After blocking, they were incubated overnight at 4 °C with primary antibody (anti-TH, anti-P-gp, anti-MPO, or anti-EBA), rinsed three times in 1× PBS containing 0.5 % BSA (10 minutes/rinse), and incubated with the appropriate biotinylated secondary antibody and avidin–biotin complex (Elite Kit; Vector Laboratories, Burlingame, CA, USA) for 1 hour at room temperature. Bound antibodies were visualized by incubating with 0.05 % diaminobenzidine–HCl (DAB) and 0.003 % hydrogen peroxide in 1× PBS. Brain sections were rinsed with 1× PBS for DAB inhibition. Immunostained cells were analyzed by bright-field microscopy.

### Immunofluorescence

Expression of Iba-1, eNOS, CD31, GFAP, AQP-4, IL-10, IL-1β, and CLN-5 was assessed using fluorescently tagged secondary antibodies. Brain sections and cocultured bEnd.3 cells were treated as already described, except that secondary antibodies included goat anti-mouse IgG (Alexa Fluor-488, green) and goat anti-rabbit IgG (Alexa Fluor-594, red). For tomato lectin (TL) labeling, sections were incubated with Alexa Fluor-594-conjugated secondary antibodies and FITC-labeled TL for 1 hour. Following secondary antibody incubation, samples were washed and mounted using a Prolong Antifade Kit (Molecular Probes, Waltham, MA, USA). Stained cells and tissues were viewed using an Olympus I × 71 confocal laser scanning microscope (Olympus, Tokyo, Japan).

### bEnd.3 cell culture

bEnd.3 cells (endothelial cell lines; ATCC, University Boulevard Manassas, VA, USA) were maintained in DMEM supplemented with 10 % FBS. Cells were cultured in 25 cm^2^ flasks and routinely passaged using trypsin–ethylenediamine tetraacetic acid (EDTA) solution. Cells were subcultured into individual wells of 24-well plates (1 × 10^5^ cells/well) or six-well plates (1 × 10^6^ cells/well) 1 day before the experiment.

### Primary astrocyte culture

Astrocyte was cultured from the cerebral cortices of mouse pups. The cortices were rinsed twice in minimum essential medium (MEM; Sigma) containing 10 % FBS and triturated mechanically. The dissociated cells were plated with 75 cm^2^ T-flasks. After 2 weeks, the astrocyte was detached from the flasks, and seeded onto 24-well plates (1 × 10^5^ cells/well) or six-well plates (1 × 10^6^ cells/well). After 30 minutes to 1 hour, the culture medium was replaced with MEM containing 5 % FBS.

### BV 2 cell culture

BV 2 cells (microglia lines; ATCC) were maintained in DMEM supplemented with 10 % FBS. Cells were cultured in 25 cm^2^ flasks and routinely passaged using trypsin–EDTA solution. Cells were cocultured into individual wells of 24-well plates (5 × 10^4^ cells/well) or six-well plates (5 × 10^5^ cells/well) in seeded astrocytes.

### VEGF-A or LPS treatment and coculture with MSCs

Seeded bEnd.3 cells were treated with VEGF-A (10 ng/ml; R&D systems, Minneapolis, MN, USA) in a humidified incubator at 37 °C for 24 hours. MSCs (3 × 10^4^ cells/well in 24-well plate or 3 × 10^5^ cells/well in six-well plate) in transwells (0.4 μm pore size; Corning, N.Y.C, NY, USA) were then placed in each well and incubated for another 24 hours. Cocultured astrocytes and BV 2 cells were treated with LPS (100 ng/ml; Sigma, Dorset, SP, USA) for 4 hours in a humidified incubator at 37 °C. MSCs (3 × 10^4^ cells/well in 24-well plate or 3 × 10^5^ cells/well in six-well plate) in transwells were then placed in each well and incubated for another 24 hours.

### Western blot analysis

Cocultured bEnd.3 cells were dissolved in ice-cold lysis buffer (20 mM Tris–HCl, pH 7.5, 1 mM EDTA, 5 mM MgCl_2_, 1 mM dithiothreitol, 0.1 mM phenylmethylsulfonyl fluoride plus protease inhibitor cocktail; Sigma). Lysates were centrifuged (20 minutes, 14,000 × *g*, 4 °C) and supernatants were transferred to fresh tubes. Proteins were analyzed using the Bio-Rad Protein Assay Kit (Bio-Rad, Hercules, CA, USA). Equal amounts of protein (20–50 μg) were loaded in each lane with loading buffer containing 0.125 M Tris–HCl, pH 6.8, 20 % glycerol, 4 % sodium dodecyl sulfate, 10 % mercaptoethanol, and 0.002 % bromophenol blue. Samples were boiled for 5 minutes before gel loading. Separated proteins were electrophoretically transferred to polyvinylidene difluoride membranes (Millipore, Bedford, MA, USA). Membranes were washed in Tris-buffered saline solution with 2.5 mM EDTA (TNE) and then blocked in TNE containing 5 % skim milk for 1 hour. Membranes were incubated overnight at 4 °C with primary antibodies (CLN-5, eNOS, IL-10, IL-1β, VEGF-A) and then were washed three times. The membranes were incubated with secondary antibody (1:10,000 dilution of horseradish peroxidase-conjugated goat anti-rabbit antibody; GenDEPOT, Barker, TX, USA) and anti-mouse antibody (GenDEPOT, Barker, TX, USA) for 1 hour at room temperature. They were developed using ECL western blotting detection reagent (Amerxham, Pittsburgh, PA, USA), followed by exposure to X-ray film. For semiquantitative analysis, the density of immunoblot bands was measured using computer imaging (Fujifilm, Tokyo, Japan).

### VEGF-A ELISA

VEGF-A levels in SN tissue were measured using a sandwich ELISA kit according to the manufacturer’s instructions (Abnova, Taipei City, Taiwan). To analyze the changes of VEGF-A levels in the midbrain following MSC treatment, we measured VEGF-A levels at 6 hours, 12 hours, 1 day, 2 days, and 7 days after LPS injection.

### Behavior test

At 1 day and 7 days after LPS injection, apomorphine-induced (0.5 mg/kg subcutaneously; Sigma) rotation was assessed for 30 minutes. Only animals showing a mean of six contralateral rotations/minute over 30 minutes following the administration of apomorphine were retained.

### Stereological cell counts

Unbiased stereological estimations of the total number of TH-positive and MPO-positive cells in the SN were made using an optical fractionator as described previously, with some modifications [15]. This sampling technique is not affected by tissue volume changes and does not require reference volume determinations [16]. The sections used for counting (eight or nine per series) covered the entire SN from the rostral tip of the pars compacta to the caudal end of the pars reticulata. Sampling was performed using the Olympus CAST-Grid system (Olympus Denmark A/S, Ballerup, Denmark) in which an Olympus BX51 microscope measured distances on the *z* axis. The SN was delineated at 1.25× magnification. A counting frame (60 %, 35 units, 650 μm^2^) was placed randomly on the first counting area and systematically moved through all counting areas until the entire delineated area was sampled. Actual counting was performed using a 40× oil objective. Guard volumes (4 μm from the top and 4–6 μm from the bottom of the section) were excluded from both surfaces, and only profiles that came into focus within the counting volume (with a depth of 10 μm) were counted. The total number of stained cells was calculated according to the optical fractionator formula [16].

### Statistical analysis

Mann–Whitney and Kruskal–Wallis tests were used to compare means between groups and for multiple comparisons, respectively. *p* <0.05 was considered statistically significant. Statistical analyses were performed using commercially available software (version 10.0; SPSS, Inc., Chicago, IL, USA).

## Results

### MSC treatment reduces BBB permeability in LPS-induced animals

**Fig. 1:**
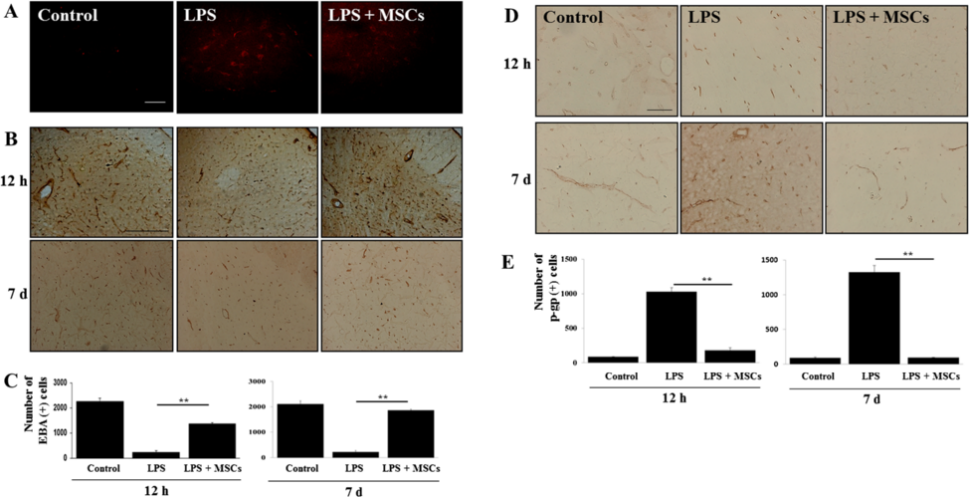
MSC treatment reduces BBB permeability in LPS-induced animals. LPS administration into the SN increased EB infiltration and decreased EBA-expressing cells; however, MSC treatment following LPS administration markedly decreased EB infiltration **a** and increased EBA-immunoreactive cells in the SN **b** (*n* = 5). **p <0.01. Scale bar: 20 μm and 100 μm. Number of EBA-positive cells as quantified by stereological analysis **c**. In addition, MSC treatment significantly decreased the number of P-gp-expressing cells in the SN of LPS-treated animals compared with only LPS-treated animals **d**, **e** (*n* = 5). **p <0.01. Scale bar: 100 μm. *EBA* endothelial-barrier antigen, *LPS* lipopolysaccharide, *MSC* mesenchymal stem cell, *p-gp* P-glycoprotein

To determine the effects of MSCs on BBB permeability, brain tissue in the SN was stained with EB, a marker for BBB permeability, and antibodies against EBA, a marker of endothelial cells. LPS administration into the SN increased EB infiltration and decreased EBA-positive cells (Fig. 1a, b); however, MSC treatment following LPS administration markedly decreased EB infiltration (Fig. 1a) and increased EBA-positive cells (Fig. 1b). Stereological analysis revealed that MSC treatment significantly increased the number of EBA-positive cells in the SN at 12 hours and 7 days after LPS injection relative to LPS-treated rats (*p* <0.001; Fig. 1c). In addition, LPS administration caused a marked increase in P-gp-positive cells (Fig. 1d); however, MSC treatment following LPS administration significantly reduced this effect (Fig. 1d). Quantification confirmed that MSC treatment significantly decreased P-gp-positive cells in the SN of LPS-treated animals compared with only LPS-treated animals at 12 hours and 7 days after LPS injection (Fig. 1e; *p* <0.001).

### MSC treatment enhances filament density in astrocytic endfeet in LPS-induced animals

**Fig. 2:**
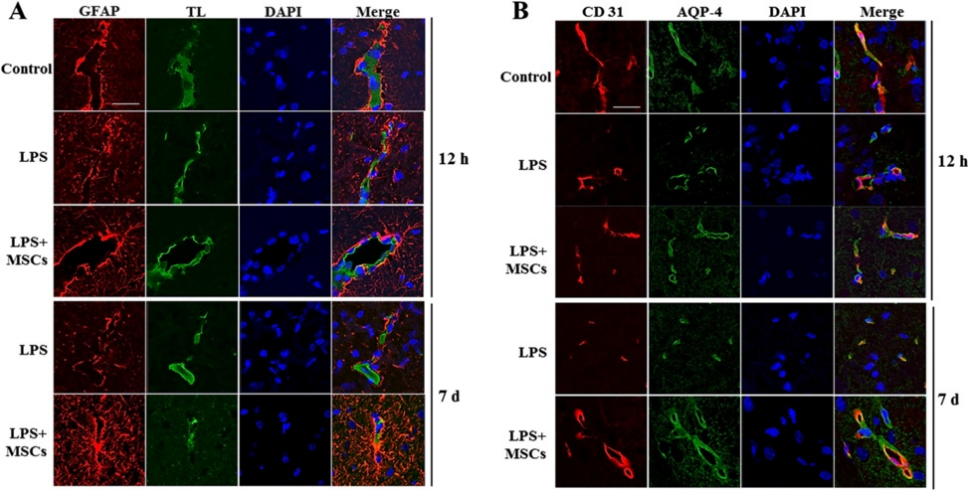
MSC treatment enhances filament density in astrocytic endfeet in LPS-induced animals. In GFAP and TL **a** or in AQP-4 and CD31 **b** double staining, astrocytic filaments and endfeet were sparsely distributed in the SN of LPS-treated animals. On the other hand, MSC treatment markedly increased the density of astrocytic filaments around vessels **a** as well as astrocytic endfeet **b** in LPS-treated animals. Scale bar: 20 μm. *AQP-4* aquaporin 4, *DAPI* 4',6-diamidino-2-phenylindole, *GFAP* glial fibrillary acidic protein, *LPS* lipopolysaccharide, *MSC* mesenchymal stem cell, *TL* tomato lectin

The astrocytic endfeet that engulf the capillary networks of the brain have been assumed to significantly influence neurovascular structure and integrity. LPS administration in the SN reduced the density of astrocyte filaments; however, MSC treatment following LPS administration markedly increased the astrocytic filament density at 12 hours and 7 days after LPS injection (Fig. 2a). To further evaluate astrocytic endfeet around vessels, GFAP and TL (Fig. 2a) or AQP-4 and CD31 (Fig. 2b) double staining was performed. Compared with controls, astrocytic filaments and endfeet were sparsely distributed in the SN of LPS-treated animals. On the other hand, MSC treatment markedly increased the density of astrocytic filaments around vessels (Fig. 2a) and astrocytic endfeet (Fig. 2b) in LPS-treated animals. The density of astrocytic filaments around vessels at 7 days after LPS injection was decreased compared with the density at 12 hours after LPS injection. However, MSC treatment increased the density of astrocytic filaments at 7 days after LPS injection relative to the density at 12 hours after LPS injection (Fig. 2a, b).

### MSCs modulate tight junction protein expression via VEGF-A in LPS-induced animals

**Fig. 3:**
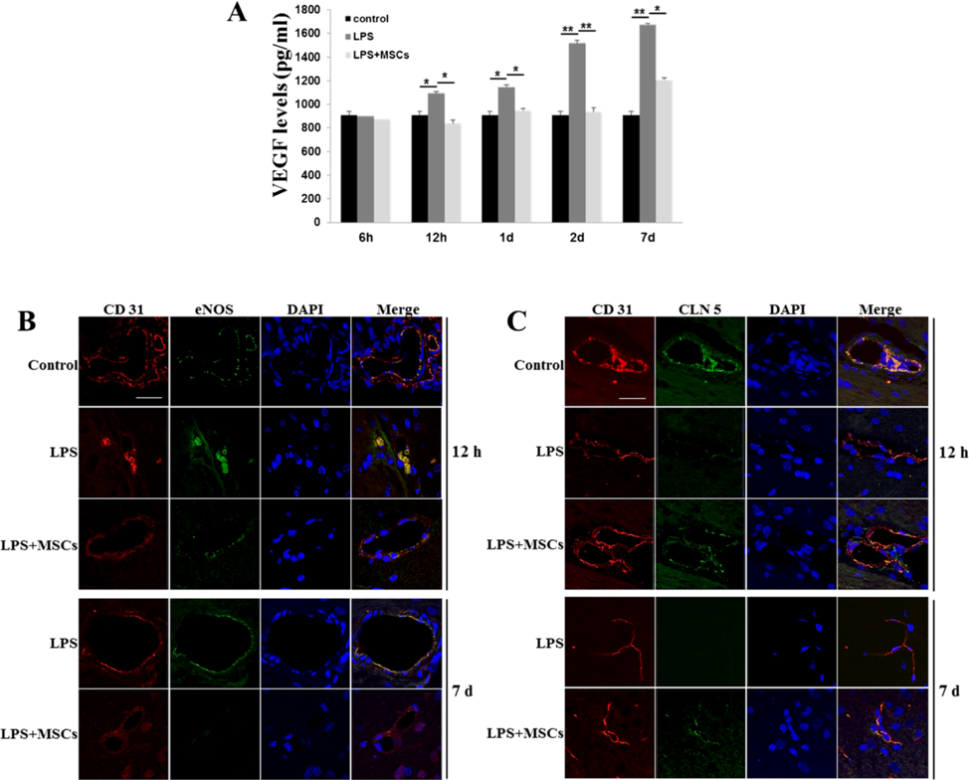
MSCs modulate tight junction protein expression via VEGF-A in LPS-induced animals. Compared with controls, the VEGF-A levels were significantly increased from 12 hours after LPS injection, and elevated VEGF-A levels were sustained 7 days after LPS injection. Meanwhile, MSC treatment significantly attenuated VEGF-A expressions in LPS-treated rats **a** (*n* = 3). *p <0.05, **p <0.01. Double immunofluorescence showed that LPS treatment markedly upregulated eNOS immunoreactivity **b** and downregulated CLN-5 immunoreactivity **c** in the endothelial cells of the SN, whereas MSC treatment attenuated eNOS expression and increased CLN-5 expression in the endothelial cells at 12 hours and 7 days after LPS injection **b**, **c**. Scale bar: 20 μm. *CLN-5* claudin-5, *DAPI* 4',6-diamidino-2-phenylindole, *eNOS* endothelial nitric oxide synthase, *LPS* lipopolysaccharide, *MSC* mesenchymal stem cell, *VEGF* vascular endothelial growth factor

To evaluate the effects of MSCs on expression of VEGF-A, we determined VEGF-A levels by ELISA at 6 hours, 12 hours, 1 day, 2 days, and 7 days after LPS injection. Compared with controls, LPS-treated rats expressed significantly greater levels of VEGF-A from 12 hours after LPS injection, and elevated VEGF-A levels were sustained 7 days after LPS injection. However, MSC treatment significantly reduced VEGF-A expression compared with that in LPS only-treated rats, and these levels were similar to those in controls (Fig. 3a). Furthermore, we evaluated whether the effects of MSCs on VEGF-A regulate eNOS expression and tight junction integrity by double immunostaining for eNOS and CD31 or CLN-5 and CD31. LPS treatment markedly upregulated eNOS immunoreactivity and downregulated CLN-5 immunoreactivity in the endothelial cells of the SN; however, MSC treatment in LPS-treated animals attenuated eNOS expression and increased CLN-5 expression in the endothelial cells at 12 hours and 7 days after LPS injection (Fig. 3b, c).

### MSC treatment reduces neutrophil infiltration and loss of dopaminergic cells in the SN

**Fig. 4:**
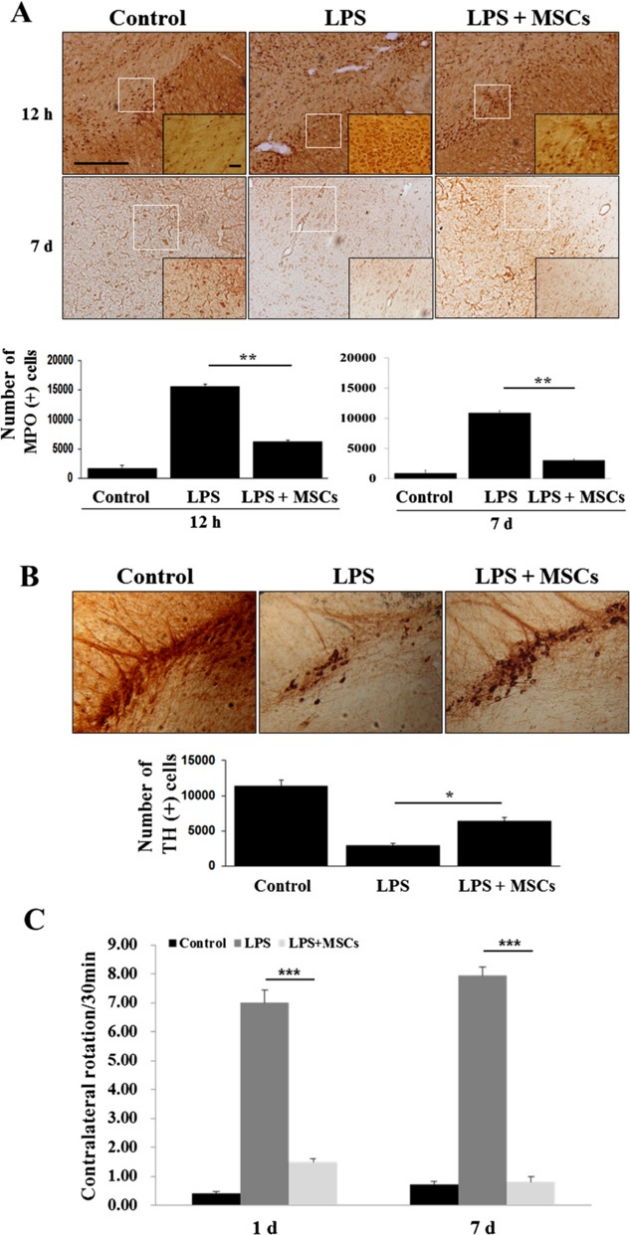
MSC treatment reduces LPS-induced neutrophil infiltration and loss of dopaminergic cells in the midbrain. There was a marked increase in MPO-positive cells in LPS-induced animals; however, MSC treatment following LPS administration notably decreased the number of these cells **a** (*n* = 5). **p <0.001. Scale bar: 100 μm. Consequently, MSC treatment inhibited LPS-induced loss of TH-positive cells in the midbrain **b** (*n* = 5). *p <0.05. Behavioral analysis showed that the number of rotations after apomorphine injection was significantly increased in animals receiving LPS compared with controls, whereas MSC administration led to a significant reduction of rotation behavior in LPS-treated animals **c** (*n* = 5). **p <0.001. Scale bar: 100 μm. *LPS*, lipopolysaccharide, *MSC* mesenchymal stem cell, *MPO* myeloperoxidase, *TH* tyrosine hydroxylase

To determine the effects of MSCs on neutrophil infiltration, SN tissue was immunostained for MPO, a marker for activated neutrophils. There was a marked increase in MPO-positive cells in LPS-induced animals; however, MSC treatment following LPS administration notably decreased these cells (Fig. 4a). Stereological analysis revealed that the number of neutrophils in the SN was significantly decreased in MSC-treated rats relative to those treated only with LPS (*p* <0.001; Fig. 4a). Consequently, MSC treatment decreased LPS-induced loss of dopaminergic neurons (Fig. 4b). On stereological analysis, the number of TH-positive cells in the SN was significantly increased in MSC-treated rats relative to that in rats treated only with LPS (*p* <0.05; Fig. 4b). Behavioral analysis showed that the number of rotations after apomorphine injection was significantly increased in animals receiving LPS compared with controls. However, MSC administration in LPS-treated animals led to a significant reduction of rotation behavior at 1 day and 7 days after LPS injection (Fig. 4c). We attempted to identify MSCs in the MSC-treated animals using human-specific NuMA immunostaining. The histological analysis showed that NuMA-positive cells were observed in the left SN (see Additional file 1).

### MSCs modulate VEGF-A-regulated tight junction protein expression in bEnd.3 cells

**Fig. 5:**
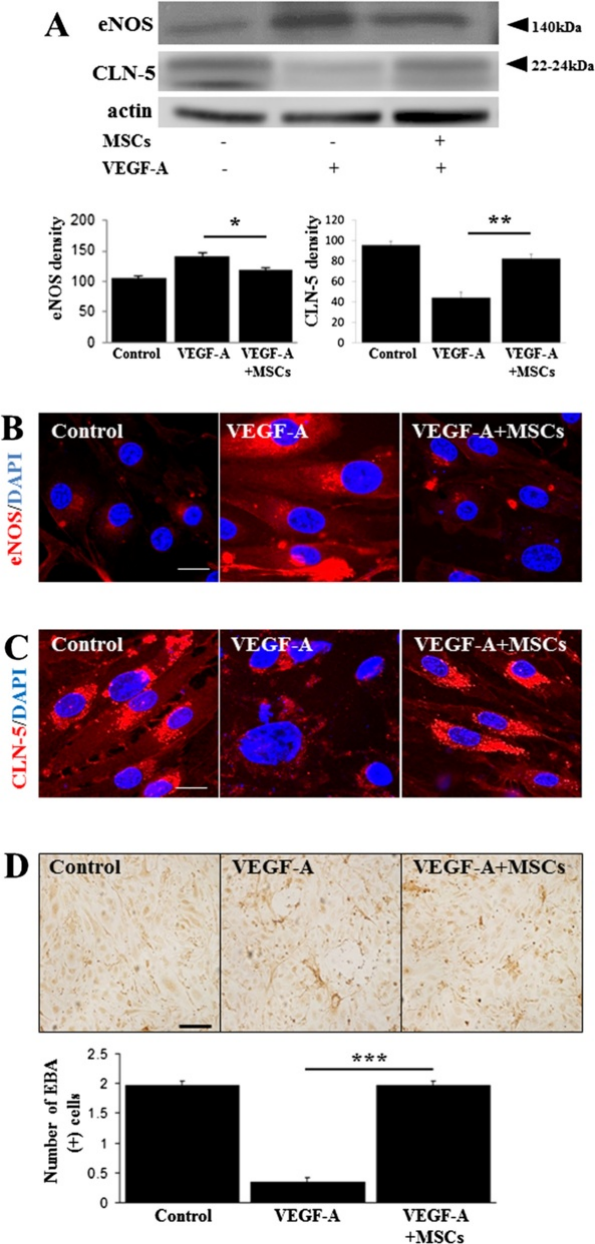
MSCs modulate VEGF-A-regulated tight junction protein expression in bEnd.3 cells. Western blots revealed that VEGF-A treatment significantly increased eNOS expression and decreased CLN-5 expression in bEnd.3 cells; however, the expressions of these proteins were reverted to control levels after MSC treatment **a** (*n* = 3). *p <0.05, **p <0.01. Immunocytochemical analysis showed that MSC treatment attenuated VEGF-A-induced increases in eNOS immunoreactivity and downregulation of CLN-5 immunoreactivity **b**, **c**. Scale bar: 20 μm. In addition, the number of detected EBA-expressing cells was significantly increased in MSC-treated cells relative to those treated only with VEGF-A **d**, **e** (*n* = 3). ***p <0.001. Scale bar: 100 μm. *CLN-5* claudin-5, *DAPI* 4',6-diamidino-2-phenylindole, *EBA* endothelial-barrier antigen, *eNOS* endothelial nitric oxide synthase, *LPS* lipopolysaccharide, *MSC* mesenchymal stem cell, *VEGF* vascular endothelial growth factor

To determine the effects of MSCs on VEGF-A-related signaling in a cellular system, endothelial cells were treated with VEGF-A and then co-cultured with MSCs. Western blots revealed that VEGF-A treatment significantly increased eNOS expression and decreased CLN-5 expression in bEnd.3 cells; however, the expressions of these proteins were reverted to control levels after MSC treatment (Fig. 5a). This result was further confirmed by immunocytochemical analysis, which showed that MSC treatment attenuated VEGF-A-induced increases in eNOS immunoreactivity and downregulation of CLN-5 immunoreactivity (Fig. 5b, c). Additionally, we assessed survival of endothelial cells after VEGF-A treatment using EBA immunocytochemistry. Whereas VEGF-A treatment decreased EBA immunoreactivity, MSC treatment in these cells markedly increased EBA-positive cells (Fig. 5d). Stereological analysis revealed that the number of detected EBA-positive cells was significantly increased in MSC-treated cells relative to those treated only with VEGF-A (*p* <0.001; Fig. 5d).

### MSCs modulate VEGF-A through anti-inflammatory action of microglia

**Fig. 6:**
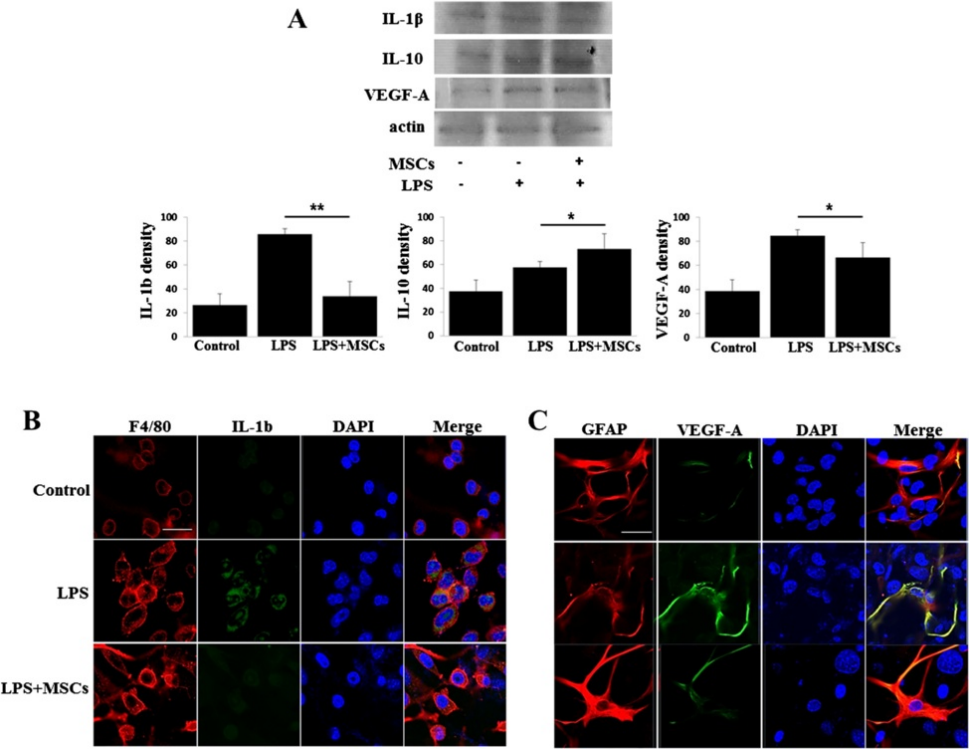
MSCs modulate VEGF-A through anti-inflammatory action of microglia. Using coculture of astrocyte and BV 2 cells, LPS treatment significantly increased IL-1β expression in BV 2 cells and VEGF-A expression in astrocytes. However, MSC treatment significantly decreased the expressions of these proteins with an increase in IL-10 levels **a** (*n* = 3). *p <0.05, **p <0.01. Immunofluorescence analysis showed that MSC treatment attenuated LPS-induced increased in immunoreactivities of IL-1β in BV 2 cells **b** and VEGF-A in astrocytes **c**. Scale bar: 20 μm. *DAPI* 4',6-diamidino-2-phenylindole, *GFAP* glial fibrillary acidic protein, *IL* interleukin, *LPS* lipopolysaccharide, *MSC* mesenchymal stem cell, *VEGF* vascular endothelial growth factor

To determine the effects of MSCs on the modulation of VEGF-A in the cellular inflammation system, cocultured astrocyte and BV 2 cells were treated with LPS and then cocultured with MSCs. Western blots revealed that LPS treatment significantly increased IL-1β expression in BV 2 cells and VEGF-A expression in astrocytes; however, the expressions of these proteins were reverted to control levels after MSC treatment (Fig. 6a). In addition, IL-10 levels in cocultured astrocytes and BV 2 cells were significantly increased after MSC treatment (Fig. 6a). This result was further confirmed by immunofluorescence analysis, demonstrating that MSC treatment attenuated LPS-induced increases in immunoreactivities of IL-1β and VEGF-A (Fig. 6b, c).

## Discussion

The present study demonstrates that MSC treatment significantly increases survival of dopaminergic neurons by stabilizing BBB permeability and attenuating neutrophil infiltration in the SN of LPS-injected animals. Furthermore, we found that MSC treatment restores astrocytic endfeet and inhibits VEGF-A-related eNOS-dependent downregulation of CLN-5 in the inflammatory conditions of cellular and animal models. The present data therefore suggest that modulation of astrocytic endfeet and astrocyte-related angiogenic factors by MSCs may represent a therapeutic strategy to stabilize BBB integrity in neurodegenerative conditions.

Ample evidence has suggested that a microglial reaction and inflammatory processes contribute to the cascade of neuronal degeneration in neurodegenerative disease [17]. Like other central nervous system diseases such as multiple sclerosis, brain cancer, or stroke, neuroinflammation is the main contributor to BBB dysfunction in neurodegenerative conditions [1, 18–20]. Several clinical studies have demonstrated BBB dysfunction in patients with neurodegenerative diseases; Skoog et al. [21] identified BBB alterations before the onset of clinical dementia in AD patients, and Kortekaas et al. [22] reported BBB impairments in restricted areas of the midbrain in patients with late-stage PD. Regarding the functional significance of BBB dysfunctions, baseline BBB integrity was closely coupled with changes in neurological deficits in patients with multiple system atrophy [23]. Furthermore, in a longitudinal study of multiple system atrophy patients, we recently demonstrated that changes in BBB integrity may be a more reliable biological marker for disease progression in neurodegenerative disease than is baseline BBB status [24]. Collectively, these in vivo data suggest that BBB integrity might modify disease progression in neurodegenerative diseases, and thus modulation of BBB integrity could be a potential treatment for neurodegenerative diseases.

The present study demonstrated that MSCs stabilized BBB alterations induced by LPS administration into the SN of rats. MSC treatment significantly restored the expression of EBA and P-gp in endothelial cells, which were significantly altered in LPS-treated animals. Consequently, MSC treatment stabilized infiltrations of EB and activated neutrophils, and thus increased the survival of dopaminergic neurons in the midbrain of LPS-treated rats. We focused our investigation on the mechanism by which MSCs modulate BBB integrity on astrocytic endfeet and astrocyte-related angiogenic factors. Astrocytes are known to be more resistant than neurons to oxidative stress [25], and they are neuroprotective because they take up potassium and glutamate and release mitogenic factors [26]. However, in the inflamed brain, astrocytes retract their endfeet from vessels, increasing BBB permeability [4–6]. More importantly, several studies have documented that expression of the angiogenic factor VEGF-A by astrocytes is increased by microglia-induced inflammatory factor IL-1β in the inflamed brain [7, 27, 28]. In turn, VEGF-A released from astrocytes binds to VEGF receptor 2 on microvascular endothelial cells and activates eNOS to downregulate expression of tight junction proteins, which may lead to disruption of endothelial tight junctions and increased BBB permeability [7, 9].

Interestingly, the mechanism by which MSCs stabilize the BBB through astrocytes seems to be complex. First, our study demonstrated that MSCs can restore the density of filaments within astrocytic endfeet surrounding microvessels. As astrocytic endfeet are a major component of the BBB, a lower density of astrocytic filaments in the midbrain is known to reduce BBB integrity in LPS-induced models of PD [29]. Restoration of astrocytic endfeet by MSCs may thus represent a morphologically stabilizing effect on BBB integrity. Second, the present study indicates that MSCs may modulate VEGF-A-related eNOS-dependent downregulation of tight junction proteins in the endothelium. In LPS-treated rats, MSC treatment decreased LPS-induced elevation of VEGF-A levels and downregulation of CLN-5. In cultured endothelial cells, employed to directly evaluate modulatory effects of MSCs on VEGF-A-related eNOS-dependent signaling, MSC treatment reduced VEGF-A-induced increases in eNOS expression and decreases in CLN-5 expression, which consequently led to increased survival of endothelial cells. Regarding the underlying mechanisms of VEGF-A-related eNOS-dependent signaling by MSCs, inflammatory cytokine IL-1β released by activated microglia would trigger this signaling pathway by binding to its receptor on the astrocyte [9]. Our previous study showed that MSCs could attenuate microglial activation though secretion of the anti-inflammatory cytokines IL-6, IL-10, and transforming growth factor beta in LPS-induced or neurotoxin-induced animal models [11]. In addition, the present study showed that, along with increased IL-10 levels after MSC treatment, MSC treatment significantly decreased the IL-1β level in BV 2 cells and the VEGF-A level in astrocytes that were elevated following LPS treatment. Accordingly, the regulatory property of microglial activation by MSCs might decrease secretion of proinflammatory cytokines into the astrocytes and then modulate sequentially VEGF-A-related eNOS-dependent signaling in the endothelium, which would stabilize expression of tight junction proteins.

The consequence of BBB alteration in parkinsonian diseases remains unclear. In neurodegenerative disease, neurons and glia are confronted with a decreased homeostatic reserve and thus become extremely sensitive and vulnerable to alterations in the extracellular milieu [30]. In turn, increased BBB permeability may increase the probability of exposing microenvironments in the brain to environmental neurotoxins, which may then interrupt mitochondrial electron transport or elements of the peripheral immune system, and subsequently accelerate neuroglial degeneration and neuroinflammation. BBB stabilization might therefore be an effective strategy to modify disease progression in neurodegenerative disease.

Whether impaired BBB integrity in neurodegenerative conditions is a compensatory response to expel toxic misfolded proteins from the brain remains unclear. Nevertheless, BBB alterations would be expected to further accelerate neuroinflammation as well as neurodegeneration [30]. Similarly, the present study showed that BBB disruption by neuroinflammation led to neutrophil infiltration in addition to dopaminergic neuronal death. However, MSC treatment restored activated neutrophil infiltrations and dopaminergic neuronal death. In this regard, regardless of the underlying response for BBB alterations, stabilizing the BBB may represent a useful therapeutic option to prevent and/or modify disease progression in PD.

## Conclusions

We have shown that MSCs stabilize BBB permeability by modulating astrocytic endfeet and VEGF-A signaling, which may be relevant as a candidate neuroprotective strategy for the treatment of neurodegenerative disease.

## Additional file

Additional file 1: Figure S1. showing histological analysis of transplanted MSCs in LPS-injected rats. The existence of MSCs in the left SN of LPS-injected rats was identified by immunohistochemistry using NuMA, a human specific marker (*white arrow*) **A**. The NuMA-positive cells were observed in animals treated with human MSCs, and the number of recruited MSCs is about 0.9 % of the total transplanted MSCs **B** (*n* = 3). (PDF 144 kb)

## Abbreviations

AQP-4: Aquaporin 4
BBB: Blood–brain barrier
BSA: Bovine serum albumin
CLN-5: Claudin-5
DAB: 0.05 % diaminobenzidine–HCl
DMEM: Dulbecco’s modified Eagle’s medium
EB: Evans blue dye
EBA: Endothelial-barrier antigen
EDTA: Ethylenediamine tetraacetic acid
ELISA: Enzyme-linked immunosorbent assay
eNOS: Endothelial nitric oxide synthase
FBS: Fetal bovine serum
FTIC: Fluorescein isothiocyanate
GFAP: Glial fibrillary acidic protein
Iba-1: Ionized calcium-binding adapter molecule 1
IL-10: Interleukin-10
LPS: Lipopolysaccharide
MEM: Minimum essential medium
MPO: Myeloperoxidase
MPTP: 1-Methyl-4-phenyl-1,2,3,6-tetrahydropyridine
MSC: Mesenchymal stem cell
NuMA: Nuclear mitotic apparatus protein
PBS: Phosphate-buffered saline
PD: Parkinson’s disease
P-gp: P-glycoprotein
SN: Substantia nigra
TH: Tyrosine hydroxylase
TL: Tomato lectin
TNE: Tris-buffered saline solution with 2.5 mM EDTA
VEGF-A: Vascular endothelial growth factor -A
