# Supplementary material for: Mesenchymal stem cells stabilize the blood–brain barrier through regulation of astrocytes
Source: Stem Cell Res Ther. 2015 Sep 29;6:187. doi: 10.1186/s13287-015-0180-4 (PMC4588687; doi:10.1186/s13287-015-0180-4)
Supplement: Additional file 1: Figure S1. — showing histological analysis of transplanted MSCs in LPS-injected rats. The existence of MSCs in the left SN of LPS-injected rats was identified by immunohistochemistry using NuMA, a human specific marker (white arrow) A. The NuMA-positive cells were observed in animals treated with human MSCs, and the number of recruited MSCs is about 0.9 % of the total transplanted MSCs B (n = 3). (PDF 144 kb) [file 13287_2015_180_MOESM1_ESM.pdf]

Additional file 1

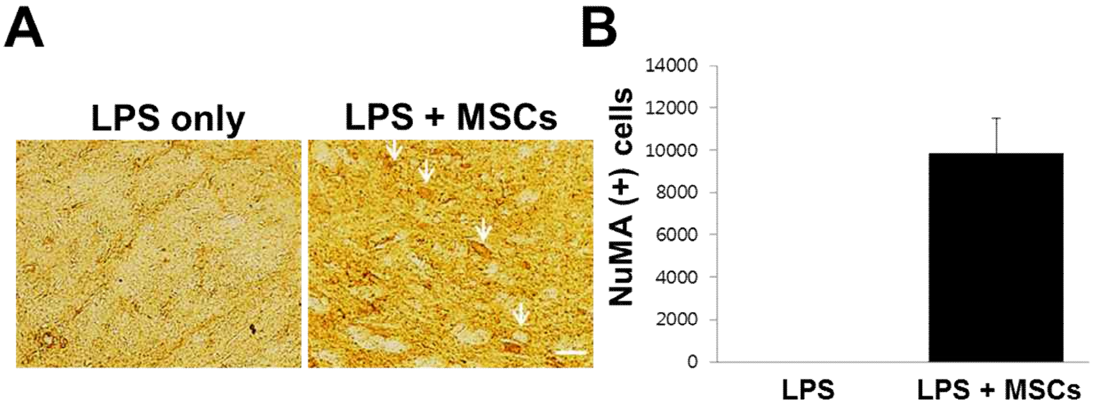

Figure S1. **Histological analysis of transplanted mesenchymal stem cells (MSCs) in LPS-injected rats.** The existence of MSCs in the left substantia nigra (SN) of LPS-injected rats was identified by immunohistochemistry using NuMA, a human specific marker (white arrow, A). The NuMA-positive cells were observed in animals treated with human MSCs, and the number of recruited MSCs is about 0.9% of the total transplanted MSCs (B, *n* = 3).
